# The Gal/GalNac lectin as a possible acetylcholine receptor in *Entamoeba histolytica*

**DOI:** 10.3389/fcimb.2023.1110600

**Published:** 2023-05-16

**Authors:** Marisol Pacheco-Sánchez, Sandra Luz Martínez-Hernández, Martín Humberto Muñoz-Ortega, Jesús Alejandro Reyes-Martínez, Manuel Enrique Ávila-Blanco, Javier Ventura-Juárez

**Affiliations:** ^1^ Departamento de Morfología, Centro de Ciencias Básicas, Universidad Autónoma de Aguascalientes, Aguascalientes, Mexico; ^2^ Departamento de Microbiología, Centro de Ciencias Básicas, Universidad Autónoma de Aguascalientes, Aguascalientes, Mexico; ^3^ Departamento de Química, Centro de Ciencias Básicas, Universidad Autónoma de Aguascalientes, Aguascalientes, Mexico

**Keywords:** *E. histolytica*, acetylcholine, Gal/GalNAc 150 kDa, virulence, receptor

## Abstract

*Entamoeba histolytica* (*E. histolytica*) is a protozoan responsible for intestinal amebiasis in at least 500 million people per year, although only 10% of those infected show severe symptoms. It is known that *E. histolytica* captures molecules released during the host immune response through membrane receptors that favor its pathogenetic mechanisms for the establishment of amebic invasion. It has been suggested that *E. histolytica* interacts with acetylcholine (ACh) through its membrane. This promotes the increase of virulence factors and diverse mechanisms carried out by the amoeba to produce damage. The aim of this study is to identify a membrane receptor in *E. histolytica* trophozoites for ACh. Methods included identification by colocalization for the ACh and Gal/GalNAc lectin binding site by immunofluorescence, western blot, bioinformatic analysis, and quantification of the relative expression of Ras 5 and Rab 7 GTPases by RT-qPCR. Results show that the Gal/GalNAc lectin acts as a possible binding site for ACh and this binding may occur through the 150 kDa intermediate subunit. At the same time, this interaction activates the GTPases, Ras, and Rab, which are involved in the proliferation, and reorganization of the amoebic cytoskeleton and vesicular trafficking. In conclusion, ACh is captured by the parasite, and the interaction promotes the activation of signaling pathways involved in pathogenicity mechanisms, contributing to disease and the establishment of invasive amebiasis.

## 1 Introduction

Amoebiasis or amoebic dysentery is an intestinal disease caused by the protozoan *Entamoeba histolytica* (*E. histolytica*), a significant cause of diarrhea in children and the third leading cause of death in the world caused by protozoa (Haque et al., 2003) infects about 500 million people per year. However, only 10% of infected patients present severe symptoms; the rest suffer an asymptomatic infection (Baxt and Singh, 2008).

The mechanism of invasion begins with the ingestion of water or food contaminated with fecal waste and cysts, moving through the esophagus until it reaches the stomach, where it softens the chitin wall of the cyst and surviving the acid pH of the stomach passes into the intestine where excystation occurs and the trophozoites migrate to the colon (Li et al., 2021).

*E. histolytica* infection is usually asymptomatic; the parasite remains in the intestinal lumen, feeding on bacteria and host cells; however, in some cases, depending on the virulence of the strain and the type of host immune response can trigger severe tissue damage promoted by the invasion process as amoebic colitis, fulminant colitis or toxic colon and liver abscesses (Taherian et al., 2022).

Over the years, evidence has been obtained to clarify the elements that cause severe infection. It is known that during the intestinal inflammatory process, *E. histolytica* trophozoites bind to the extracellular matrix (ECM) such as collagen, laminin, and fibronectin (FN) (Sengupta et al., 2009) for example the lectin 140 kDa for FN (Flores-Robles et al., 2003), the lectin 220 kDa for hyaluronic acid (Meza et al., 2006) and lectin 170 kDa for collagen (Petri et al., 2002).

In response, the immune system releases molecules such as IL-1β, IL-6, IL-8, IL-12, interferon-gamma (IFN-γ), tumor necrosis factor-alpha (TNF-α), and prostaglandin E2 (García-Zepeda et al., 2007). Previous studies have shown that interferon-gamma (IFN-γ) binds on the surface of *E. histolytica* through a membrane receptor of approximately 200 kDa; as a result, it stimulates the expression of its virulence factors (Pulido-Ortega et al., 2019). Diaz-Valencia et al. (2015) reported that *E. histolytica* is responsive to IL-8 through a 29 kDa membrane receptor. When IL-8 binds to the trophozoite membrane, it activates signaling pathways that induce a reorganization of the amoeba cytoskeleton, which promotes the ability of *E. histolytica* to migrate to the inflammation zone (Diaz-Valencia et al., 2015). *E. histolytica* is known to secret prostaglandin E2-like protein that stimulates IL-8 mRNA expression in host epithelial cells (Dey and Chadee, 2008) and modifies cell permeability by affecting claudin-4 conforming tight junctions claudin-4 (Lejeune et al., 2011).

Acetylcholine (ACh) has also been reported as a modulator of *E. histolytica* virulence; it is the main neurotransmitter in the colon and, during the inflammatory process, is released by the enteric autonomic nervous system to regulate the immune response, studies carried out with trophozoites stimulated with exogenous ACh evidenced a significant increase in virulence mechanisms such the adhesion, synthesis and secretion of C amebapores, cysteine proteinase 2 (*ehcp-a2*) and cysteine proteinase 5 (*ehcp-a5*); moreover, in the model of amoebic liver abscess in hamsters, an excellent progression of lesions was shown (Medina-Rosales et al., 2021).

The molecular mechanism(s) by which the amoeba exogenously uptakes ACh and activates signaling cascades to regulate these virulence factors is unknown, so this work aimed to identify the possible molecular mechanism of ACh uptake in *E. histolytica* trophozoites.

## 2 Methodology

### 2.1 Axenic culture of *E. histolytica*

The HM-1: IMSS strain was cultured under axenic conditions in TYI-S-33 culture medium, supplemented with 10% Fetal Bovine Serum (FBS) (Microlab, Mexico), penicillin (100 U/ml) and streptomycin (100 mg/ml) at 37°C (Diamond et al., 1978).

### 2.2 Immunofluorescence assay

The trophozoites (1×10^6^) were placed on glass coverslips in the bottom of each well of a 24-well with TYI-S-33 and incubated for 15 min at 37°C, ACh 0.01 µM was added, and the plate was further incubated for 1 h at 37°C. Trophozoites were washed with PBS-1X, fixed with 2% paraformaldehyde, and blocking was performed with 2% Fetal Bovine Serum (FBS); the permeabilization was with 0.2% Triton X-100 in PBS-1X. For the double immunodetection, trophozoites were treated with anti-acetylcholine-FITC polyclonal antibody (1:800; LifeSpan BioSciences LS-C305726, Seattle, Washington, USA) or primary anti-Gal/GalNAc monoclonal antibody (1:1000; Ab00446-10.0-BT, Oxford, UK), incubated for 1 h, then washed with PBS-1X and further incubated with secondary antibody Alexa Fluor 594 conjugated goat anti-mouse IgG (H+L) (1:1000; Invitrogen A-11005, Eugene, Oregon, EE. UU.). Cells were washed with PBS-1X and incubated with 1 μg/ml Hoechst 33342 (Sigma‐Aldrich, Poole, Dorset, UK) for nuclear staining, finally, washed and coverslips with Vectashield (Vector Laboratories, Burlingame, California, USA) and were observed with a Carl Zeiss LSM 700 Laser Scanning Microscope (Carl Zeiss AG). Images were acquired using the Zen Black 2012 (black edition) software (ZEISS). Images were analyzed with ImageJ software (Wayne Rasband, Nat. Inst. Of Health, USA) to identify ACh and Gal/GalNAC colocalization. The comparative degree of colocalization was calculated as mean Spearman’s and Mander’s R coefficients, considering R values above 0.6 threshold values as significant. The same procedure described above was performed for the Gal/GalNac lectin inhibition assays; 200 mM of N-acetylgalactosamine was used (McCoy et al., 1994).

### 2.3 Protein modeling and ligand

The amino acid sequence in FASTA format of the intermediate subunit of the Gal/GalNAc lectin belonging to *E. histolytica* was obtained from the UniProt database with the accession code C4LV76. The sequence was modeled using the I-TASSER server (Yang et al., 2015). The ligands used for molecular docking with the Gal/GalNAc lectin intermediate subunit were ACh (ID: 187) and N-acetylgalactosamine (ID: 35717); both compounds were obtained from the PubChem database.

### 2.4 Molecular docking

Protein processing consisted of binding polar hydrogens, merging nonpolar hydrogens, and calculating Kollman’s charge with AutoDock Tools v.1.5.6 (Morris et al., 2009). Ligand preparation consisted of adding hydrogens under pH 7.4, minimizing their energies under an MMFF94 force field employing Avogadro v.1.2.0 software; subsequently, Gaisteiger charges were calculated with AutoDock Tools v.1.5.6. With the DoGSiteScorer tool of the ProteinPlus server (Schöning-Stierand et al., 2020) the protein binding regions were predicted, only those regions with a druggability score greater than or equal to 0.75 were evaluated. Molecular docking was performed using AutoDock v.4.2 software; 64 X 64 X 64 boxes were used, covering individually the previously predicted regions.

### 2.5 Protein-ligand interactions

The best conformations obtained for each binding site were subjected to Protein-Ligand interaction analysis, where hydrophobic interactions and hydrogen bridges formed between protein residues and ligands. The PoseView tool of the ProteinPlus online server was used to obtain the interactions.

### 2.6 RT-qPCR

An RNA pool of 1 × 10^6^ trophozoite controls and trophozoites incubated with 0.01 µM ACh was obtained with the Direct-zol RNA Miniprep kit (Zymo Research, Irvine, California, USA), following the manufacturer’s protocol and subsequently evaluated for purity and concentration using the BioDrop (Isogen Life Science, Barcelona, Spain).

Reverse transcription was performed with 500 ng of RNA using the Revert Aid First Strand cDNA Synthesis Kit (Thermo Fisher Scientific California, USA). For gene expression quantification, 50 ng of cDNA was used *via* real-time quantitative PCR with Maxima SYBR Green qPCR Master Mix (2×) (Thermo Scientific, California, USA) in a Step One thermal cycler (Applied Biosystems, Thermo Fisher Scientific, California, USA) with the following conditions: 50˚C for 2 min and 95˚C for 3 min, followed by 40 cycles of 95˚C for 45 sec and 56˚C for 45 sec. Relative expression levels were normalized with the housekeeping gene alpha-tubulin, and differences were determined using the 2^-ΔΔCq^ method (**Table 1**).

**Table 1:** Primers directed to the *E. histolytica* gene were used in this study.

Target	Primers Sequence 5´3´	Access numbers
Tubulina	Fwd:TGC ACC AAT TGT TAC ACC AGA	XM_648327.1
	Rev: CAT GGA CAC CAT CCA ACA AA	
Ras 5	Fwd: TGC ATC AGG AGC AAT AGG AA	XM_00191425.1
	Rev: CCC TGA AAT TTT GCC TTT CTT	
Rab 7	Fwd: TTG GTG ATT CAG GTG TTG GA	XM_644104.2
	Rev: CGT TCA TTT CCG GCA GTA TC	
Gal/GalNAc lectin heavy subunit 170 kDa	Fwd: TGC ACA TGT CCA ATG TGT TG	M59850.1
	Rev: CAC TTT TGG TTG GCA TGT GT	
Gal/GalNac lectin intermediate subunit 150 kDa	Fwd: TGATGAGTGCGAAGATGGTT	XM_649881.2
	Rev: TGAGCAACGGCTTCAGTACA	

### 2.7 Immunoprecipitation assays

The trophozoites (1×10^6^) were incubated in TYI-S-33 medium for 15 min at 37°C, ACh 0.01 µM was added, and incubation continued for 1 h at 37°C. Subsequently, the trophozoites were washed three times with PBS-1X to remove excess ACh. Trophozoites without interaction with ACh were also prepared as a control. Trophozoites with or without acetylcholine were homogenized and lysed in RIPA buffer (Sigma-Aldrich, St Louis, Missouri, USA) with protease inhibitor cocktail (Merck, Darmstadt, Germany) at 4°C for 30 min. The lysates were centrifuged at 4000 ×*g* for 10 min at 4°C, and one part of the supernatant was separated (total lysate). 3 μg of anti-Gal/GalNAc monoclonal antibody (Ab00446-10.0-BT, Oxford, UK) was added to the rest of the supernatant and incubated overnight at 4°C with slow shaking. Subsequently, these antibody-treated cellular lysates were added 10 μL of protein A agarose beads and incubated for 2 h at 4°C. The beads were collected by centrifugation and washed three times with lysis buffer. Protein elution was followed by immunoblot analysis

### 2.8 Western blot

For Western blotting, 30 µg of the protein extract was separated from the total lysate on a 10% SDS-PAGE gel, and the immunoprecipitation products were also placed on this gel, proteins were transferred to polyvinylidene difluoride (PVDF) membranes (Bio-Rad, Philadelphia, PA, USA). The membranes were blocked with Tris-buffered saline (TBS) and 5% skimmed milk for 1 h at room temperature. For immunodetection, the membranes were incubated overnight at 4°C with anti-Gal/GalNAc monoclonal antibody (1:1000; Ab00446-10.0-BT, Oxford, UK) or anti-acetylcholine polyclonal antibody (1:1000; LifeSpan BioSciences LS-C739212, Seattle, Washington, USA). Blots were incubated for 2 h at room temperature with goat anti-mouse IgG-HRP conjugated (1:5,000; AP127P, Chemicon, USA) or goat anti-rabbit IgG-HRP conjugated (1:5,000; A0545, Sigma, USA). After the incubation, the membranes were washed with TBST-1X (Tris-buffered saline– 0.05% Tween 20) and revealed with Clarity Western ECL substrate (Bio-Rad, Hercules CA, USA) for chemiluminescence imaging.

## 3 Results

### 3.1 Colocalization of Gal/GalNAc lectin and ACh

To identify ACh uptake sites in *E. histolytica* trophozoites, we evaluated the involvement of the amoebic lectin Gal/GalNAc, a molecular heterotrimer that recognizes sugars such as galactose (Gal) and N-acetylgalactosamine (Petri et al., 2002) and ECM components such as collagen and FN (Sengupta et al., 2009). This amoebic cell surface protein is composed of a heavy subunit (Hgl 170 kDa), a light subunit (Lgl 31 kDa) covalently linked by an intermediate subunit (lgl 150 kDa) (Pacheco et al., 2004) with protein tyrosine kinase activity (Hernández-Ramírez et al., 2000).

To determine whether Gal/GalNAc lectin can act as a receptor for ACh, we used a mouse IgG1 anti-Gal/GalNAc first antibody (1:1000; Ab00446-10.0, Absolute Antibody Cleveland USA) and a goat-anti-mouse IgG Alexa Fluor 594 (H+L) secondary (1:1000; Invitrogen A-11005, Eugene, Oregon, USA), which identifies the anti-lectin antibody, concomitantly we applied an anti-acetylcholine-FITC antibody (1:800; LifeSpan BioSciences LS-C305726, Seattle, Washington, USA) that recognizes ACh on the trophozoite membrane.

**Figure 1:**
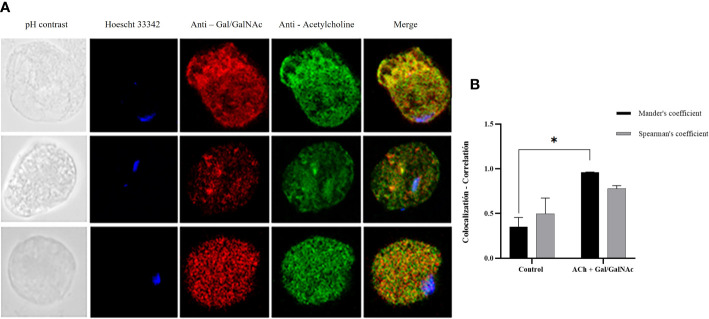
Colocalization of ACh and Gal/GalNAc lectin. **(A)** Trophozoites treated with 0.01 µM ACh for one hour. They were fixed and permeabilized. For double immunodetection, anti-Gal/GalNAc (1:1000), and anti-acetylcholine-FITC (1:1000) Hoechst 33342 (blue) antibodies were used for nuclear staining. Images were obtained with confocal microscopy (40X). **(B)** Colocalization between ACh and Gal/GalNac Lectin quantified and compared using Spearman’s and Mander’s correlation coefficients (considering values above the threshold of 0.6 as significant R coefficients). Data correspond to the mean ± SEM of five independent experiments (n = 5). Statistical analysis was performed with the one-way ANOVA method and Tukey’s post-test, where values of *p < 0.05 were considered significant.

In the image analysis, we observed colocalization between Gal/GalNAc lectin and ACh; the image analysis showed through Mander’s coefficient that the fluorescent markers (independent) colocalize in the same area of the cell, while Sperman’s coefficient showed that these sites correlate 0.98 of the pixels, so we can infer that the site acting as a collector for ACh is the amoebic Gal/GalNAc lectin (**Figure 1**).

**Figure 2:**
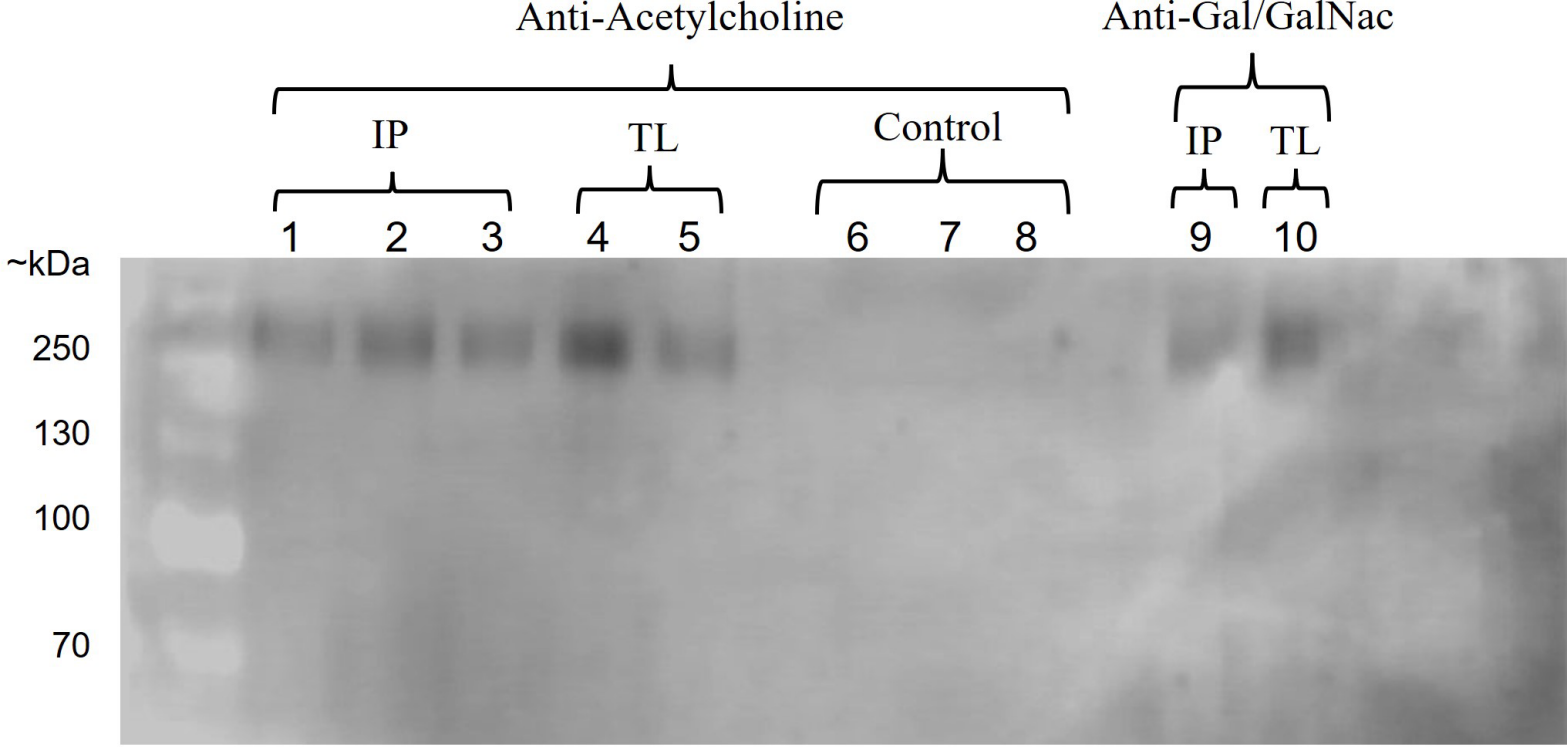
Interaction of ACh with Gal/GalNAc lectin. Western blot of immunoprecipitated proteins from *E. histolytica* using anti-acetylcholine and anti-Gal/GalNAc antibodies. Positive immunostaining was observed in proteins obtained from trophozoites interacting with ACh using an anti-acetylcholine antibody (IP: immunoprecipitated proteins, lane: 1-3, TL: total lysate, lane: 4-5) and anti-Gal/GalNAc antibody (IP: immunoprecipitated proteins, lane: 9, TL: total lysate, lane: 10). In contrast, no positive immunostaining was observed for proteins obtained from trophozoites that do not interact with ACh (control, lane: 6-8).

To confirm these findings, we performed an immunoprecipitation assay of proteins from *E. histolytica* trophozoites that interacted or did not with ACh and obtained using the anti-Gal/GalNAc monoclonal antibody. The proteins were analyzed by Western blot using both anti-Gal/GalNAc monoclonal and anti-acetylcholine polyclonal antibodies. Our results show that when we used the anti-acetylcholine antibody on the proteins obtained from trophozoites that interacted with ACh, we obtained positive immunolabeling (~ 260 kDa) (IP: immunoprecipitated proteins, TL: Total Lysate). On the other hand, when we used the anti-Gal/GalNAc antibody on the same proteins (IP: immunoprecipitated proteins, TL: Total Lysate), we also observed positive labeling (~ 260 kDa). Finally, for proteins obtained from non-interact trophozoites with ACh, we did not observe positive immunolabeling when using the anti-acetylcholine antibody on both IP and TL samples.”. These results indicate that the ACh-capturing protein is of the same molecular weight as the Gal/GalNAc immunolabeled lectin (**Figure 2**).

**Figure 3:**
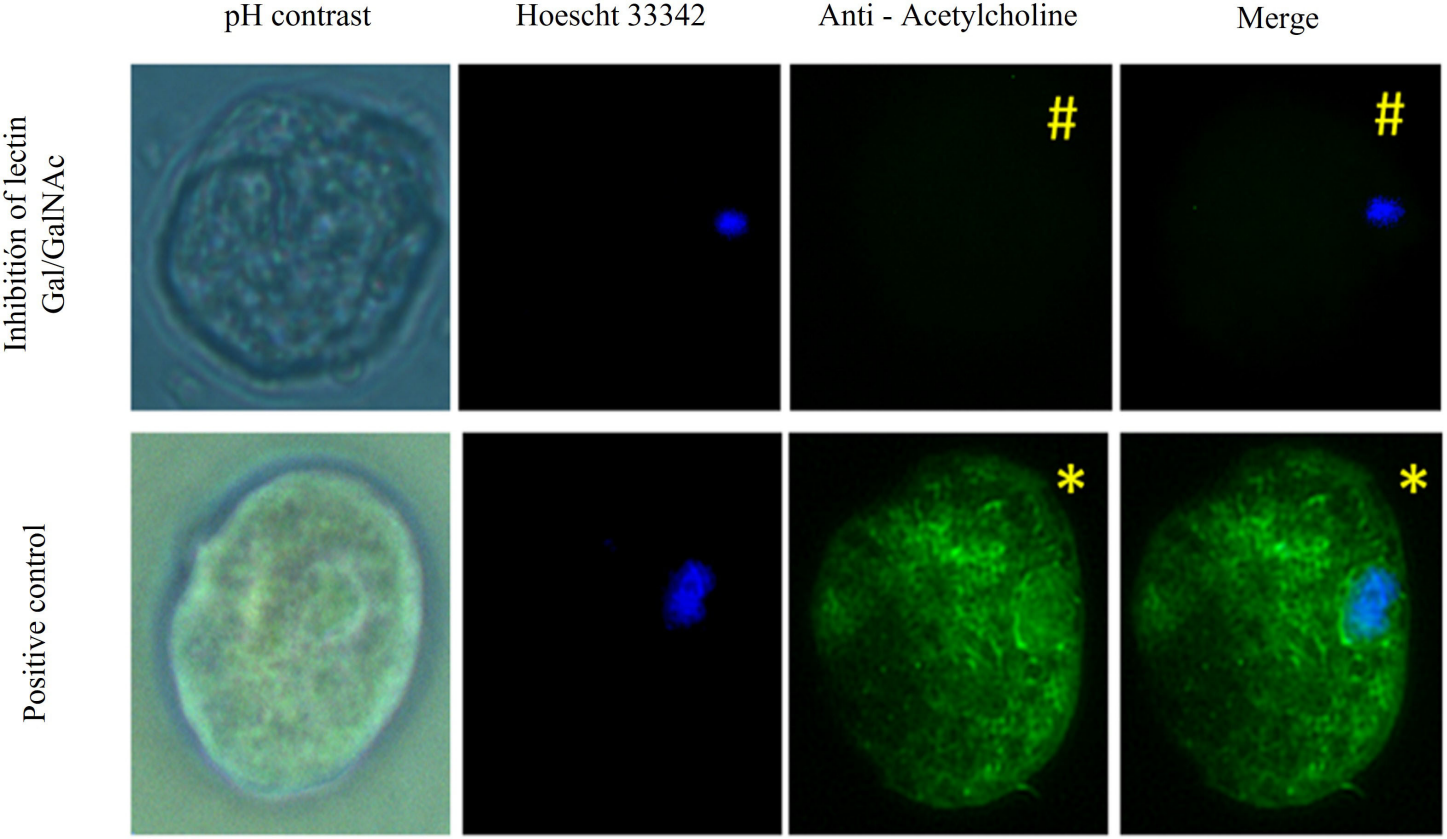
Gal/GalNAc receptor blocked with N-acetylgalactosamine. Trophozoites were inhibited with N-acetylgalactosamine and treated with 0.01 µM ACh for one hour. They were fixed and permeabilized. Anti-acetylcholine-FITC (green) (1:1000) antibody Hoechst 33342 (blue) were used for nuclear staining and photographed by EPI fluorescence microscopy (Axioskop 40, Zeiss) at 40x. We observed that the positivity to ACh in the trophozoite membrane was notably decreased in which a pretreatment was applied with N-acetylgalactosamine (#) compared to trophozoites that only interacted with ACh (*).

On the other hand, we assayed for inhibition of Gal/GalNAc lectin with N-acetylgalactosamine (McCoy et al., 1994). We applied ACh 0.01 µM for 1 hour and identified this neurotransmitter with the anti-acetylcholine-FITC antibody. We observed that the positivity to ACh in the trophozoite membrane was notably decreased (#) compared to trophozoites that only interacted with ACh (*) (**Figure 3**).

### 3.2 Molecular docking

To determine the role of the Gal/GalNAc lectin as a receptor for ACh, molecular docking was performed using bioinformatics tools widely used to predict possible ligand-protein binding sites (**Figure 3**), the results showed that the binding site between ACh and the intermediate subunit (150 kDa) of the Gal/GalNAc lectin, four binding sites (SU1, SU2, SU3, and SU4) (**Table 2**) of ACh were predicted, only those regions with a druggability score greater than or equal to 0.75 (Aretz et al., 2014) as a positive control, the assay was performed by interacting this lectin with its ligand N-acetylgalactosamine. The first one (SU1) had a score of 0.8, being the highest of all and having a volume of 911.74 Å3. The second (SU2) we obtained a score of 0.79 and a volume of 869.72 Å3. The third binding site (SU3) results showed a druggability score of 0.76 and a volume of 683.55 Å3. Finally, the fourth binding site (SU4) had a score of 0.75 and a volume of 513.12 Å3 (**Table 3**).

**Table 2:** Predicted binding sites for the Gal/GalNAc lectin intermediate subunit obtained by the DoGSiteScorer tool of the ProteinPlus server.

Binding Site	Drugability	Volume (Å^3^)	Area (Å^2^)
SU1	0.80	911.74	1303.08
SU2	0.79	869.72	1315.88
SU3	0.76	683.55	1168.48
SU4	0.75	513.12	875.93

Only those binding sites with a value equal to or greater than 0.75 in the druggability score were used.

**Table 3:** Results of the molecular couplings between the intermediate subunit of the Gal/GalNAc lectin and the ligands ACh and N-acetylgalactosamine obtained with the AutoDock Tools v.1.5.6 program for each of the predicted binding sites.

Ligand	Binding Site	Energy binding (Kcal/mol)	RMSD	Amino acids
Acetylcholine	SU1	-5.14	128.90	Val232
	SU2	-5.66	163.26	Glu101
	SU3	-4.69	163.65	Glu69
	SU4	-6.01	137.71	Ile265
N-acetylgalactosamine	SU1	-4.54	140.16	Tyr323, Tyr195, Cys191, Glu236 y Lys182
	SU2	-4.10	172.98	Asn79, Gln49 y Gln81
	SU3	-4.38	158.32	Lys67, Glu157, Asp151 y Thr159
	SU4	-4.16	142.57	Glu320, Asn297 y Met311

**Figure 4:**
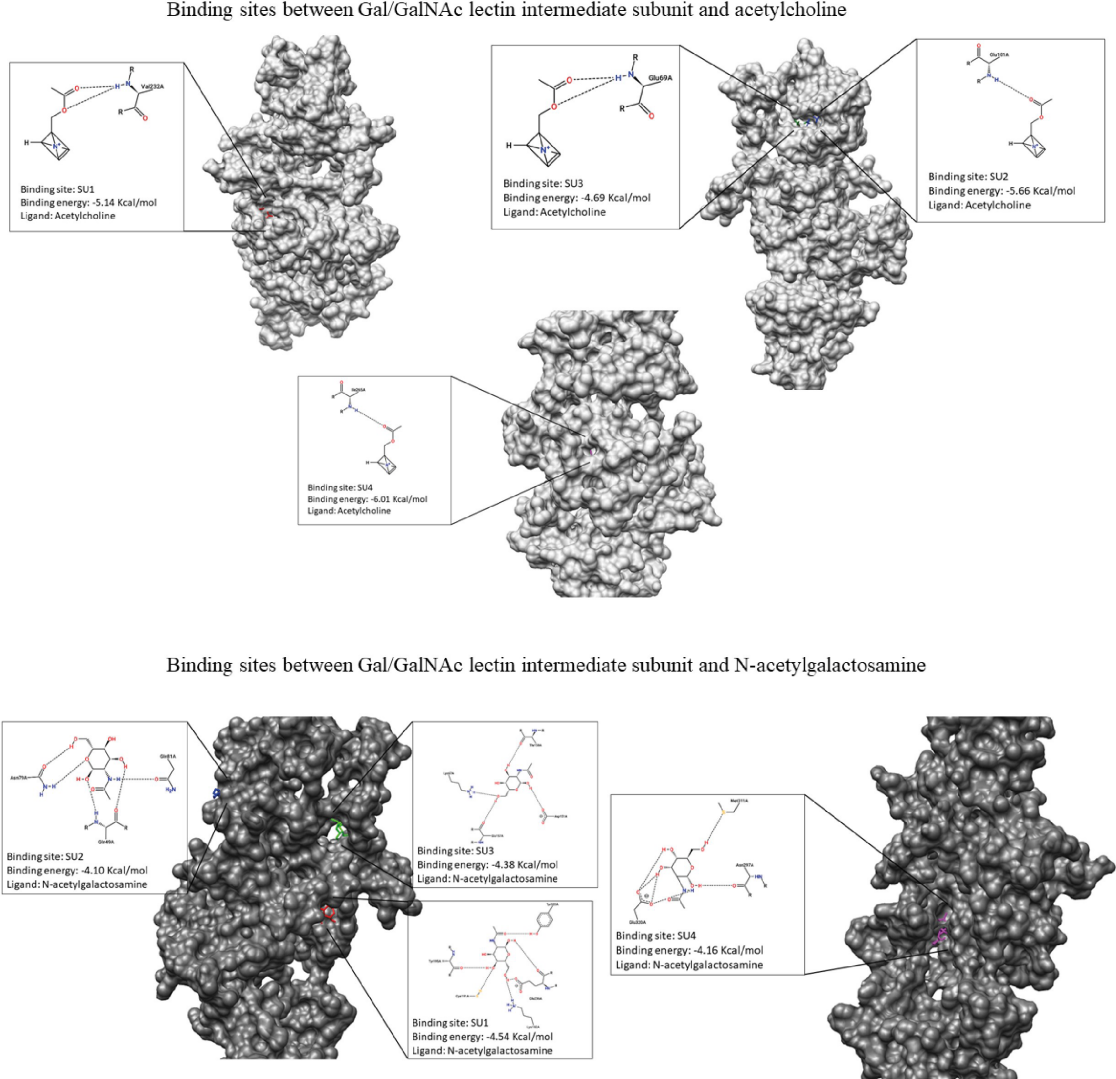
Representation of molecular dockings results obtained by Autodock and PoseView respectively. Interactions between GalGal/NAc lectin intermediate subunit and acetylcholine are shown in gray. Interactions between GalGal/NAc lectin intermediate subunit and N-acetylgalactosamine are shown in dark gray.

We then interacted with these 4 possible binding sites (SUs) with ACh; the results indicated that for SU1, the binding energy was -5.14 Kcal/mol, showing an interaction with the residue Val232. In the case of SU2, the binding energy was -5.66 Kcal/mol and presented an association with the Glu101 residue. For SU3, the interaction occurred with the amino acid Glu69 with a binding energy of -4.69 Kcal/mol. Finally, SU4 displayed a binding energy of -6.01 Kcal/mol, and the interaction was with Ile265 (**Table 3**; **Figure 4**).

For molecular docking between N-acetylgalactosamine and the intermediate subunit of the lectin Gal/GalNAc, SU1 yielded a binding energy of -4.54 Kcal/mol and interacted with residues Tyr323, Tyr195, Cys191, Glu236, and Lys182. The binding energy obtained for SU2 was -4.10 Kcal/mol, with interactions with residues Asn79, Gln49, and Gln81. SU3 had a binding energy of -4.38 Kcal/mol, and interactions occurred with amino acids Lys67, Glu157, Asp151, and Thr159. Finally, SU4 showed a binding energy of -4.16 Kcal/mol and interactions with Glu320, Asn297, and Met311 (**Table 3**; **Figure 4**).

**Figure 5:**
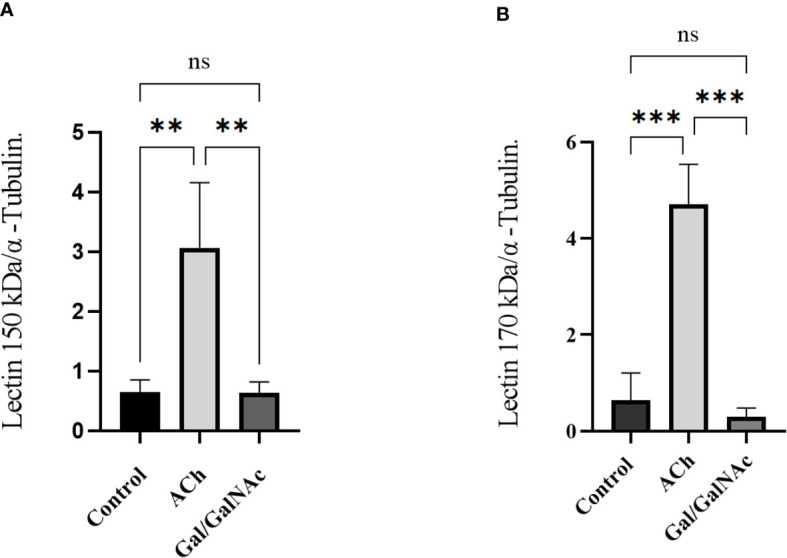
Relative expression of trophozoites under basal conditions. (Control), stimulated with 0.01 µM ACh, and with lectin inhibited by N-acetylgalactosamine (Gal/GalNAc). **(A)** Lectin Gal/GalNAc heavy subunit (170 kDa). **(B)** Lectin intermediate subunit (150 kDa) by RT-qPCR amplification of trophozoites treated with 0.01 µM ACh for 1 hour. Relative expression levels normalized to constitutive α-tubulin gene. Statistical analysis was performed with the one-way ANOVA method and Tukey’s posttest, where values of ** p < 0.01, ***p < 0.001 were considered significant and non-significant values are shown as: ns.

The relative expression of genes coding for the intermediate and heavy subunit of the Gal/GalNAc lectin (150 and 170 kDa) (**Table 1**) important participants in amoebic pathogenesis was evaluated; expression levels were normalized to the α-tubulin gene. It has been reported that the relative expression of these subunits increases after interaction with exogenous ACh (Medina-Rosales et al., 2021). Furthermore, trophozoites inhibited at the Gal/GalNAc lectin binding sites with N-acetylgalactosamine and interacted with ACh did not express the 170 kDa and 150 kDa genes compared to the control; instead, trophozoites interacted directly with ACh did show an increased relative expression of the Gal/GalNAc lectin genes (150 and 170 kDa) (**Figure 5**).

### 3.3 ACh stimulates vesicular trafficking in *E. histolytica* through Ras and Rab

It is a known fact that the intermediate subunit (lgl 150 kDa) of the Gal/GalNAc lectin shares similarity with the amino acid sequence of receptor tyrosine kinases (Tyrk), and these receptors trigger the mitogen-activated protein kinase pathway where Ras and Rab gene expression has been identified and are both involved in signaling-dependent processes (Verma et al., 2018).

Since our results showed a possible Gal/GalNAc lectin interaction with ACh, we were interested in identifying possible signaling pathways activated after exposure to ACh.

**Figure 6:**
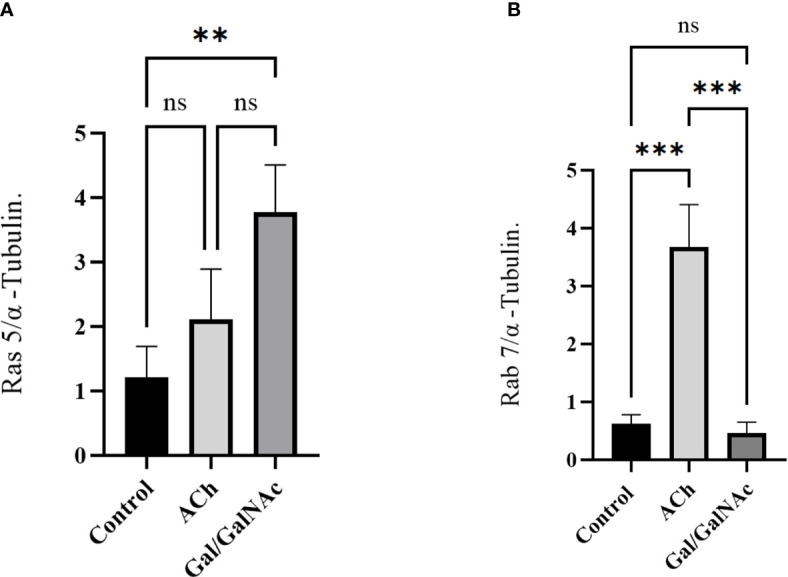
Relative expression of Ras 5 and Rab 7 in trophozoites treated with ACh by RT-qPCR. Treated trophozoites with 0.01 µM ACh and with lectin inhibited by N-acetylgalactosamine (Gal/GalNAc). **(A)** Stimulation with ACh did not increase Ras 5 expression. **(B)** In contrast, a significant change in Rab 7 expression was observed. Relative expression levels were normalized using the constitutive gene α-tubulin. Statistical analysis was performed with the one-way ANOVA method and Tukey’s posttest, where values of ** p < 0.01, ***p < 0.001 were considered significant and non-significant values are shown as: ns.

The results suggest that Ras and Rab’s expression increased significantly after 1 hour of ACh exposure; meanwhile, in trophozoites where Gal/GalNAc lectin was inhibited, a deficient expression of Rab 7 was observed, while the expression level of Ras 5 increased significantly (**Figure 6**).

## 4 Discussion

During the intestinal invasion of *E. histolytica*, a pro-inflammatory process is triggered in which IL-8 expression increases; subsequently, a TH2-type cellular and humoral response is established leading to the invasion of the parasite (Nakada-Tsukui and Nozaki, 2016). This inflammatory process is regulated by the parasympathetic autonomic nervous system releasing ACh, ACh promotes a decrease in the production of proinflammatory cytokines by inhibition of the transcription factor NF-κB and activation of anti-inflammatory cytokines through the JAK2-STAT3 signaling pathway (de Jonge et al., 2005; Altavilla et al., 2006). ACh is the key neurotransmitter in the large intestine, thus constantly innervating it to regulate glandular contractions and secretions (Eskandari et al., 2003). Therefore the presence of ACh at the site of infection can affect the elimination of the parasite from the body. In a previous study, our research group determined that the interaction between ACh and *E. histolytica* causes an increase in its virulence factors such as adhesion, synthesis, and secretion of amebapore C, and cysteine proteinase 2 (*ehcp-a2*) and cysteine proteinase 5 (*ehcp-a5*) and a significant increase in amebic liver abscess in hamsters (Medina-Rosales et al., 2021).

In this work, through structural studies, morphological analysis, gene expression, protein-ligand interaction, Western blot, and molecular bioinformatics, we have established that *E. histolytica* responds to ACh through the Gal/GalNAc lectin. It’s possible that this interaction occurs through the intermediate subunit (Igl), and that signaling pathways related to the activation of G proteins, which promote the increase of Ras 5 and Rab 7 GTPases, are also involved in this interaction.

The amoebic Gal/GalNac lectin was characterized as the first amoebic receptor that binds to the glycoproteins galactose (Gal) and N-acetylgalactosamine (GalNAc) that are found in the mucin layer of the host and on the surface of colonic epithelial cells (Petri et al., 2002) as well as being involved in cytotoxicity processes, avoiding the action of the complement system, participating in the encystation process and producing the cyst wall (Frederick and Petri, 2005).

In this work, we found one more function for the Gal/GalNAc lectin, the recognition of exogenous ACh, which is consistent with other described molecules; Villalobos-Gomez et al (Petri et al., 2002), established that the amoebic Gal/GalNAc lectin induces the activation of macrophages and neutrophils inducing an early pro-inflammatory effect, process modulated by neurotransmitters such as ACh and adrenaline, this confirms the capacity of the Gal/GalNAc lectin to respond to neurotransmitters and to adapt the conditions for the establishment of the amebic infection (Ventura-Juárez et al., 2009).

Likewise, the intermediate subunit (Igl) of the Gal/GalNAc lectin has been recognized for its tyrosine kinase activity and its functionality as a receptor for glycosylated proteins such as fibronectin (FN) (Petri et al., 2002), collagen (Talamas-Rohana and Meza, 1988), and laminin (Cheng et al., 2001).

In amoebic pathogenesis, cell adhesion is a decisive factor in tissue dissemination. Trophozoites bind to the ECM *via* membrane receptors that interact with molecules released by the host and, in turn, activate signaling pathways in the parasite that activate phosphokinase C or adenylyl cyclase, which induces the reorganization of the actin cytoskeleton (Meza, 2000), especially the intermediate subunit of the Gal/GalNAc lectin works as a receptor for fibronectin (Sengupta et al., 2009) this interaction involves the mobilization of internal vesicles to the cytoplasmic membrane (Hernández-Ramírez et al., 2007) carried out by Rab 7 GTPases, which induce the rearrangement of the cytoskeleton and vesicular transport (Javier-Reyna et al., 2012).

**Figure 7:**
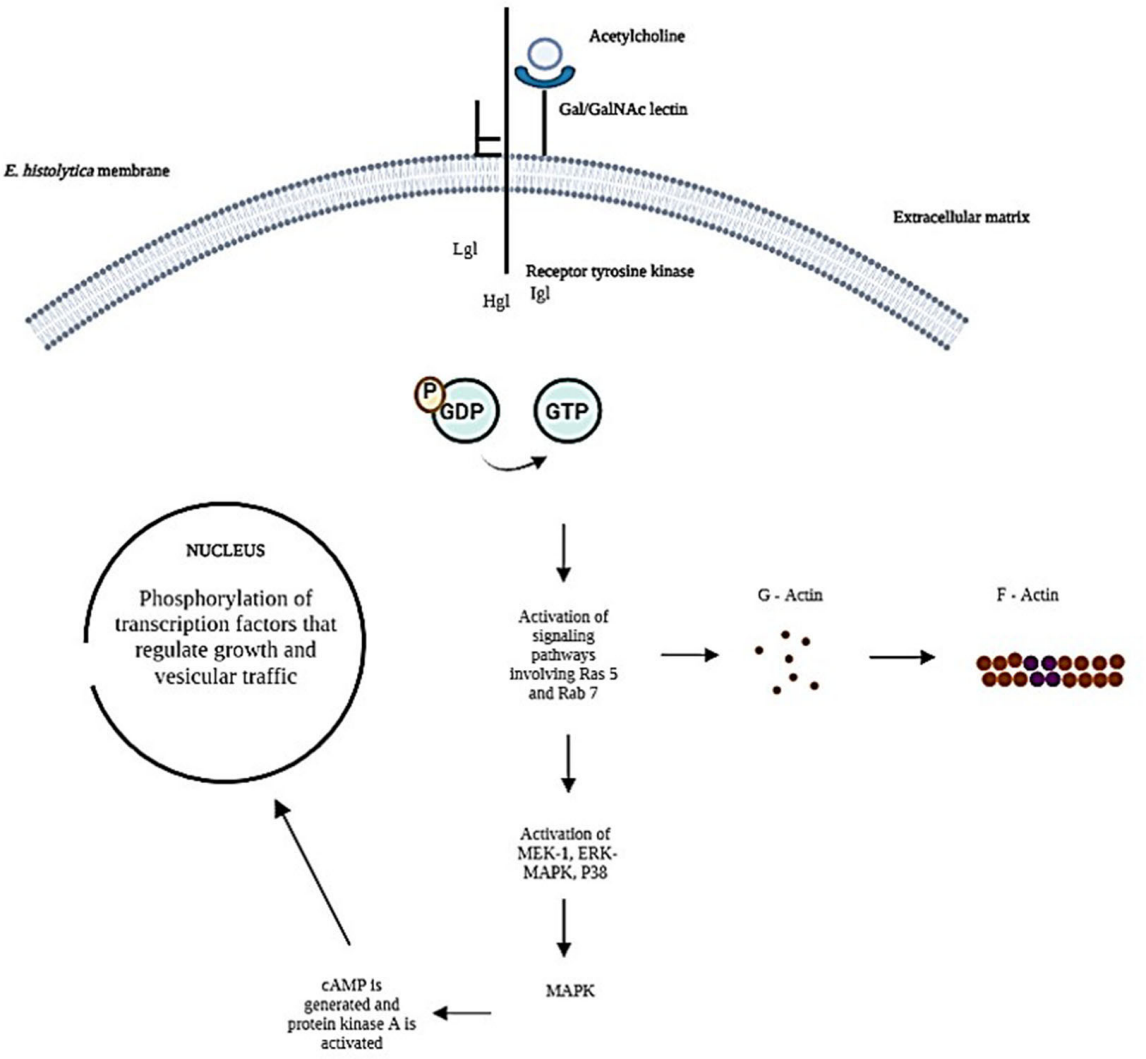
Representing the possible signaling pathways activated during the interaction of trophozoites with exogenous ACh has been taken up by the Gal/GalNAc lectin intermediate subunit receptor.

To explain the possible mechanism by which *E. histolytica* trophozoites bind ACh to their membrane and cause effects at the subcellular level. The intermediate subunit of tyrosine kinase-like receptors (Meza, 2000) and a signal peptide domain in the extracellular space (Hernández-Ramírez et al., 2007) possesses the necessary structure to bind exogenous ACh. In contrast, the heavy subunit of the Gal/GalNAc lectin contains only a transmembrane domain (Hernández-Ramírez et al., 2007), thus establishing a flow of information from the extracellular matrix to the interior of the parasitic cell (**Figure 7**).

Assuming that ACh is taken up by the intermediate subunit of the Gal/GalNAc lectin while the heavy subunit is activating the signaling pathway receptor-mediated signal transduction induces changes in cytoskeleton reorganization that contribute to cell proliferation and vesicular transport (Hernández-Ramírez et al., 2000) our results indicate an increase in Ras and Rab GTPases.

The publication of the complete genome of *E. histolytica* reported the presence of genes encoding for heterotrimeric G protein (Gα, Gβ, and Gγ) and small GTPases belonging to the Ras superfamily (Loftus et al., 2005); our group had an interest in evaluating the expression of GTPases Ras 5 and Rab 7, proteins involved as molecular mediators in processes such as cell proliferation, cytoskeleton reorganization and vesicular membrane transport (Verma et al., 2018) in addition to ECM receptor-mediated signal transduction.

The mechanism by which G proteins in *E. histolytica* are activated is still unclear (Javier-Reyna et al., 2012); however, our results suggest the participation of G proteins during the interaction of trophozoites with acetylcholine; we evaluated the expression of Rab 7, a protein that has been identified as one of the most important GTPases in the signal transduction pathway since it regulates fundamental amebic processes for parasite survival, such as phagocytosis and endocytosis, which depend on the rearrangement of the cytoskeleton and vesicular transport (Loftus et al., 2005; Naiyer et al., 2019). ACh interaction with trophozoites significantly increased Rab 7 expression and the inhibition of Gal/GalNAc lectin with its ligand N-acetylgalactosamine affected the expression, a similar effect was observed by Emmanuel et al (2015), where he reported that *E. histolytica* trophozoites upon interaction with N-acetylgalactosamine inhibited the expression of Rab 5, 7 and 21, as well as the activity of the cytoskeleton. On the other hand, ACh did not stimulate a high expression of Ras 5 compared to Rab 7, and its increase was only observed when the Gal/GalNAc lectin interacted with its ligand, these results indicate that ACh does not participate as significantly in the activation of signaling pathways mediated by Ras 5 (Naiyer et al., 2019), this *in vitro* assay may resemble the adhesion that occurs during host-parasite interaction, causing overexpression of Ras 5 GTPases (Naiyer et al., 2019), this protein controls cell proliferation, metabolism, and cell survival. In addition, Gal/GalNAc lectin, after binding to host epithelial cells, activates signaling cascades in the parasite, starting with the phosphorylation of GDP to GTP (Ikawa et al., 1980); they subsequently activate kinases such as MEK-1, ERK-MAPK, and p38; these cytokines induce the expression of the mitogenic pathway, leading to increased cell proliferation and differentiation (Ikawa et al., 1980).

In our results, the participation of the Gal/GalNAc lectin in ACh uptake could be increasing Rab 7 levels, which possibly stimulated conformational changes of the cytoskeleton and increased vesicular traffic that led to greater secretion of amoebapore C, cysteine. proteinase and increased adhesion to host epithelial cells (Medina-Rosales et al., 2021).

Overexpression Rab 7 suggests a molecular mechanism that may resemble what could happen during binding between fibronectin and *E. histolytica* trophozoites that involve activation of G-like proteins leads to the phosphorylation of transcription factors that would regulate the vesicular transport.

These results strongly suggest that a host with acetylcholine levels close to 0.01 µM in the intestine is more likely to have a severe amoebic infection, normal blood acetylcholine levels are known to be 8.66 ± 1.02 pmol/ml in the blood, and 3.12 ± 0.36 pmol/ml in plasma while in the large intestine (Kawashima and Fujii, 2000) there is a concentration of 8.51 +/- 3.15 nmole/g (Ikawa et al., 1980) as can be seen, it is a concentration very close to that used during the experiment, so in the face of an imbalance in intestinal homeostasis and an increase in the concentration of acetylcholine, you may be susceptible to developing the amoebic infection.

## 5 Conclusion

Using confocal microscopy, Western blot, gene expression, and bioinformatics studies, we observed a possible ACh binding in the membrane of *E. histolytica* trophozoites *via* the amoebic lectin Gal/GalNAc, this possible interaction could induce conformational changes of the cytoskeleton and increased vesicular trafficking, resulting in a significant increase in Rab 7 and stimulation of Ras 5.

## Data availability statement

The original contributions presented in the study are included in the article/supplementary material. Further inquiries can be directed to the corresponding author.

## Author contributions

PSM paper writing, design of experiments, analysis of experiments, discussion of results. MHSL design of experiments, analysis of experiments, discussion of results. MOMH design of experiments, analysis of experiments and discussion of results. ABME paper design. RMJA figure making. VJJ manuscript revision, results revision, and discussion revision. All authors contributed to the article and approved the submitted version.
